# Patterns of Psychotropic Prescribing Practices in Autistic Children and Adolescents: An Australian Perspective of Two Cohorts Five Years Apart

**DOI:** 10.1007/s10578-024-01710-5

**Published:** 2024-06-01

**Authors:** Anna Baldes, Tamara May, Amanda Brignell, Katrina Williams

**Affiliations:** 1 Department of Paediatrics, Monash University, Clayton, VIC Australia; 2 Mental Health, Drugs and Alcohol Services, Barwon Health, Geelong, VIC Australia; 3 Murdoch Children’s Research Institute Parkville, Parkville, VIC Australia; 4 Developmental Paediatrics, Monash Children’s Hospital, Clayton, VIC Australia; 5 Department of Paediatrics, University of Melbourne, Parkville, VIC Australia

**Keywords:** Autism spectrum disorder, Autism, Drug utilisation, Psychotropic drugs, Australia

## Abstract

This study aims to describe the utilisation of psychotropic medications in Australian autistic children and adolescents. All children and adolescents with available Pharmaceutical Benefits Scheme data who endorsed an autism diagnosis in The Longitudinal Study of Australian Children, including both B (n = 233, age 0–1 years in wave 1) and K cohorts (n = 157, age 4–5 years in wave 1), were included to describe psychotropic prescribing patterns. 212 (54.4%) autistic children and adolescents received at least one psychotropic prescription and 99 (25.4%) had polypharmacy. The most common psychotropic class prescribed was antidepressants (31.3%). Children in the B cohort were more likely to have a parent-reported diagnosis of anxiety or depression (χ^2^ = 12.18, p < 0.001) and tended to be more likely to have received a psychotropic prescription (χ^2^ = 3.54, p = 0.06). Psychotropic prescribing in Australian autistic children is common despite limited evidence for efficacy and tolerability of psychotropics in this group.

## Introduction

Psychotropic prescribing in autistic children and adolescents is common [1, 2]. This is despite medication having not been shown to be effective in changing core autistic social communication characteristics. Several potential agents have been proposed, including oxytocin and the cholinergic and glutamatergic agents currently used in Alzheimer’s dementia, these remain experimental [3–7]. As such, there are no Australian pharmacological guidelines for the treatment of autism. International and Australian guidelines regarding psychotropic prescribing in the broader population of children and adolescents take into account the potential impact of psychotropic medications on the developing brain and limited evidence for efficacy, with recommendations that all psychotropics should be used with caution in children and adolescents [8, 9]. Psychotropic agents are also used to support psychological symptoms and co-occurring conditions; including severe behavioural disorders, attention-deficit/hyperactivity disorder (ADHD), anxiety and depression [1]. In Australia, risperidone is the sole listed medication under The Therapeutic Goods Administration (TGA) and funded by the Pharmaceutical Benefits Scheme (PBS), a branch of Australia’s universal health insurance scheme which supplements medication costs, for the management of ‘severe behavioural disturbances’ in individuals with a diagnosis of autism [10]. The prescribing criteria specify that the treatment must be commenced prior to 18 years of age under the supervision of a paediatrician or psychiatrist for ongoing severe aggression and injuries to self or others despite non-pharmacological measures being in place [10]. In a previous study, using the same cohort of Australian children as the present study, found that 31% of children with autism were prescribed a psychotropic agent as opposed to 3% where there was no diagnosis of autism [2].

High prevalence of prescribing likely reflects the heterogeneity that exists in cohorts of autistic children, which are difficult to account for in guidelines. Several factors have been associated with the use of psychotropics in autistic children and adolescents. Prescribing has been reported to be higher in females than males [11]. Autistic children who have parent-reported higher severities of autism have also been reported to be more frequently prescribed psychotropics [2]. Less frequent prescribing occurs in families with lower income status and lower parental education [12].

High prevalence of co-occurring medical, developmental and psychiatric conditions also influence prescribing. A large observational study in the UK, conducted using an extensive primary care database, including over 20 000 individuals with autism, found co-occurring anxiety and depression occurred in 15.2% and 11.0% respectively, ADHD 14.3%, and epilepsy 4.5% [11]. This has also been reported in prescribing in Australia, with a previous study utilising the same population as the present study finding that psychotropics were supplied to 74% of children and adolescents where there was autism with co-occurring ADHD, compared to 22% of children and adolescents with autism alone [2]. The prevalence and patterns of antidepressant prescribing, including agents that are also known to treat anxiety, in autistic children is less understood despite the high prevalence of co-occurrence. There have been trials of selective serotonin reuptake inhibitors (SSRIs) in children with autism with the rationale that these may address repetitive behaviours, given they are the mainstay treatment in obsessive–compulsive disorder (OCD) [13, 14]. Though evidence remains insufficient for a direct benefit in autistic children, these medications may be indicated in addition to psychological therapy if there is co-occurring anxiety or depression, with the greatest evidence of effectiveness being for fluoxetine in the broader child and adolescent population [15]. However, consideration is also needed regarding potential adverse effects, including of worsening of problematic behaviours, such as agitation, through activating effects, and discomfort from gastrointestinal side effects. How prescription rates have been affected by recommendations, co-occurring anxiety and depression and as individuals become older has not been explored.

### Aims

The primary aim of this study was to describe the utilisation of psychotropic medications in autistic children and adolescents (hereafter referred to collectively as children) in Australia using linked data from The Longitudinal Study of Australian Children (LSAC) and the PBS; extending on previous work [2] which looked at a single time point by including all available data collection time points (waves). The number of children prescribed psychotropic medications and age of first prescriptions was investigated along with potential changes in prescribing practices over time, and as the children got older. Potential changes in patterns of prescribing and diagnosis are explored by comparing the two available cohorts aged 5 years apart.

The secondary aim of this study was to provide a more detailed description of the use of antidepressants, which also include first line pharmacotherapeutic agents for anxiety disorders, in this cohort given that these appear to be commonly prescribed however not necessarily indicated. Whether fluoxetine was used as the first-line antidepressant agent was assessed, given its higher efficacy and inclusion in child and adolescent guidelines [15, 16]. Additionally, the relation between timing of autism diagnosis and first antidepressant prescription was explored.

## Methods

### Data Sources

LSAC is a longitudinal prospective population-based study following the development of a representative sample of around 10 000 Australian children [17]. Beginning in 2003, two cohorts of children and their families, the B cohort comprising 0–1 year-old infants and K cohort comprising 4–5 year-old preschool children, were surveyed every two years (called waves). Consent for linkage with Medicare Australia, including PBS data, was provided by 97% of parents of children included in LSAC and linkage was successful in 93% of children [17].

PBS data details information that is related to subsidised prescriptions and is provided continuously from the first prescription to the most recent release of LSAC data in 2020.

### Study Population

Given the understanding of autism as an enduring neurodevelopmental condition, we included all children and adolescents where PBS data was available and there was a parent reported autism diagnosis, regardless of wave, from both B cohort (n = 233) and K cohort (n = 157). Parents were asked by interview: “Does ‘study child’ have any of these ongoing conditions? (‘Ongoing conditions’ exist for some period of time (weeks, months or years) or re-occur regularly. They do not have to be diagnosed by a doctor.) Autism, Asperger’s, or other autism spectrum”. If the parent had answered yes to this question, we classified the child or adolescent as having autism. This question has been used in previous studies to define the presence of autism [2, 18, 19]. In wave 8 the K cohort study adolescent was surveyed directly responding to the question: “Do you have any of these ongoing conditions? Autism, Asperger’s, or other autism spectrum.” If yes was answered to this question, they were also classified as having autism and included in the study. The age the study child was diagnosed with autism was reported based on the earliest wave where parent reported survey data was available specifying this. Where autism diagnosis was only endorsed by the K cohort in wave 8 there was no equivalent survey question regarding age of diagnosis. In these instances (n = 16), the age of diagnosis was assumed to be 18 years old. Regarding diagnosis of anxiety or depression at waves 4 to 8 parents were asked: “Does child have any of these ongoing conditions? (Ongoing conditions’ exist for some period of time (weeks, months or years) or reoccur regularly. They do not have to be diagnosed by a doctor.) Anxiety disorder, Depression.” From wave 6 onwards, two separate survey responses were allowed with parents able to specify “Anxiety disorder” or “Depression” separately or both. As with autism diagnosis, K cohort study adolescents were invited to respond at wave 8. For our purposes, responses for anxiety and depression were combined and percentages of children with a parent reported or self-reported diagnosis of depression or anxiety at any point were described. Survey data was collected and used in the same way for ADHD and epilepsy or seizure disorders. The study child’s sex was reported based on parent survey responses in wave 1.

### Psychotropic Medication

Prescription data were available from 1st May 2002 to 31st March 2019. Consistent with a previous study using LSAC data [2], psychotropic medications were defined by the World Health Organization (WHO) ATC group N category (Table 1). The number of children prescribed these classes per year were reported, as well as the rates of polypharmacy, defined as a child receiving two or more classes of psychotropic agents within 365 days. Percentages of children prescribed psychotropics and ages of first prescriptions, using the date the prescription was supplied and child date of birth as reported in wave 1 were described. When comparing between cohorts, prescriptions were limited to prescriptions occurring between 4 and 14 years old, as prescription data beyond 14 years old is not available for the B cohort currently and prescription data is not available prior to 4 years old for the K cohort.

**Table 1:** Psychotropic classes

Anticonvulsants	Antidepressants	Antipsychotics	Anxiolytics	Hypnotics	Clonidine	ADHD medications
Carbamazepine Clonazepam Lamotrigine Levetiracetam Oxcarbazepine Phenobarbital Phenytoin Pregabalin Sulthiame Topiramate Sodium valproate Zonisamide	Amitriptyline Citalopram Desvenlafaxine Duloxetine Escitalopram Fluoxetine Fluvoxamine Imipramine Mirtazapine Nortriptyline Paroxetine Reboxetine Sertraline Venlafaxine	Aripiprazole Chlorpromazine Lithium Lurasidone Olanzapine Pericyazine Quetiapine Risperidone	Alprazolam Diazepam	Temazepam	Clonidine	Atomoxetine Dexamphetamine Methylphenidate

Psychotropic classes were defined as per the World Health Organisation ATC group N category. Only agents that were prescribed for autistic children in the data are listed

Focusing on antidepressant prescribing, the number of children prescribed each antidepressant agent per year by cohort, age at first prescription and frequency of fluoxetine being used first-line were described. Additionally, the percentage of children with autism diagnosis reported prior to first antidepressant prescription and timeframe between diagnosis and first prescription of an antidepressant were calculated by cohort using the date of first supply and age of autism diagnosis, as defined above.

### Statistical Analysis

Descriptive statistics including mean, standard deviations (SD), ranges, frequencies and medians (Mdn) with interquartile ranges (IQR) for nonparametric data were reported. All calculations were performed using StataSE v17 [20] or Excel 2019. P-values were calculated using t-tests or chi-squared tests for comparing means or Mann Whitney tests for non-parametric data, determined via Shapiro–Wilk test. To reduce the risk of false positives due to multiple comparisons, a Bonferroni correction was utilised with p < 0.003 required to reach significance. Survival analysis was undertaken for the age of first antidepressant prescription using a Kaplan Meier Curve with ages in years converted to whole integers prior (eg. 5.00–5.99 = 5).

## Results

### Demographics

**Fig. 1:**
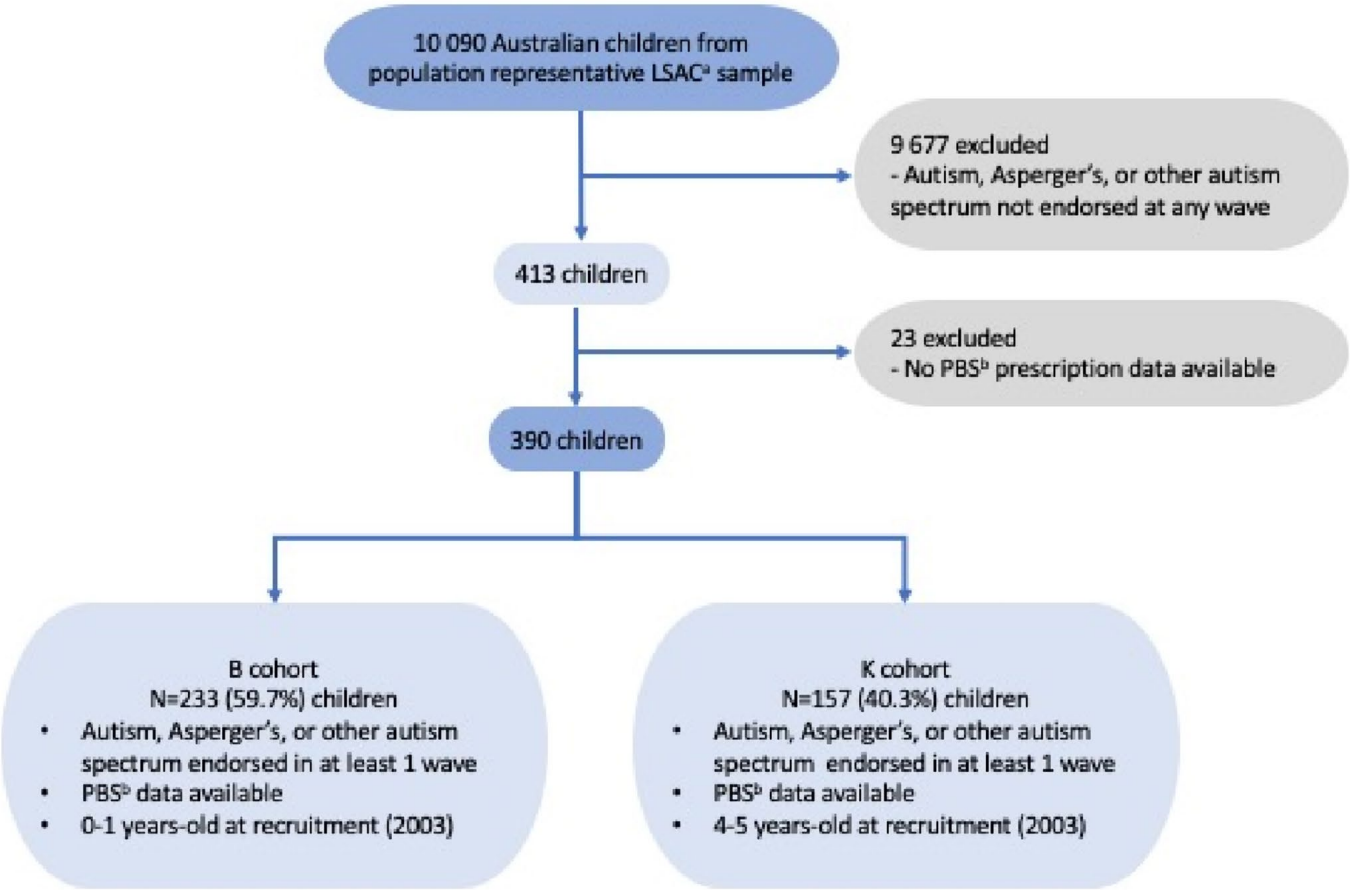
Participant selection process. ^a^The Longitudinal Study of Australian Children. ^b^Pharmaceutical Benefits Scheme

A total of 413 children were identified to have endorsed a diagnosis of autism on at least one wave during the study period. Of these, 23 children (B cohort n = 16, K cohort n = 7) had no PBS data available, psychotropic or otherwise. This may represent declined consent to linkage with Medicare, failed data linkage or, less likely, that they did not receive any prescriptions, psychotropic or otherwise, throughout the study period. For the purposes of this study these children were excluded (Fig. 1). Of the excluded 23 children, 18 or 78.3% were male and the median age of autism diagnosis was 5 years (IQR = 3—7 years).

Of the included 390 children (B cohort n = 233, K cohort n = 157), 297 or 76.2% were male and the median age of autism diagnosis was 6 years (IQR = 3–9 years), with no significant difference between cohorts, p = 0.92 and 0.47 respectively. Most (70.8%) children in the B cohort endorsed a diagnosis of anxiety or depression compared to 53.5% of children in the K cohort; χ^2^ = 12.18, p < 0.001. The percentage of children with reported ADHD and epilepsy or seizure disorder were similar in both cohorts with overall 35.6% (p = 0.84) and 5.9% (p = 0.75) endorsing a diagnosis respectively.

### Psychotropics

In total, 212 (54.4%) children received at least one psychotropic prescription during the study period, whilst polypharmacy occurred in 99 (25.4%) children. The number of children prescribed a given class per year for B and K cohorts is presented in Table 2 and 3, respectively. Antidepressants and ADHD medications were the most frequently prescribed, with 122 (31.3%) and 115 (29.5%) children receiving these respectively. ADHD medication prescriptions consisted of stimulants, prescribed in 107 (27.4%) children, and atomoxetine, prescribed in 21 (5.4%) of children. Hypnotics were the least utilised (7 children). As expected, the number of children prescribed psychotropics increased over time as the children aged, noting the impact of data only being available for the beginning of 2019.

**Table 2:** Number of children prescribed psychotropic classes per year—B cohort (N = 233)

Psychotropic Classes^b^	Age range (yrs)^a^/Year																		Total children^c^
		0–1		2–3		4–5		6–7		8–9		10–11		12–13		14–15		16–17	
	2002	2003	2004	2005	2006	2007	2008	2009	2010	2011	2012	2013	2014	2015	2016	2017	2018	2019	
Anticonvulsants	N/A	0	1	2	4	6	4	5	4	5	5	6	7	8	5	6	3	4	16
Antidepressants	N/A	0	0	0	0	2	2	5	3	8	10	14	17	19	20	28	29	23	56
Antipsychotics	N/A	0	0	0	0	1	2	9	10	11	11	14	7	8	9	8	8	4	28
Anxiolytics	N/A	0	0	0	0	0	0	1	0	0	4	0	1	1	1	3	1	0	10
Hypnotics	N/A	0	0	0	0	0	0	0	0	0	0	0	0	1	0	0	0	0	1
Clonidine	N/A	0	0	0	1	2	2	7	6	7	6	9	9	9	11	9	5	1	27
ADHD medications	N/A	0	0	0	0	4	4	9	23	36	37	45	40	43	35	38	28	21	72
Polypharmacy	N/A	0	0	0	0	5	5	9	11	15	12	17	15	17	21	25	16	11	49
Total children^d^	N/A	0	1	2	5	10	10	23	33	52	60	69	64	68	56	62	53	40	120

^a^Approximate ages are extrapolated from LSAC recruitment parameters, namely; recruitment occurred between 2003 and 2004 with the B cohort being comprised of infants between 0 and 1 years

^b^If a given child is prescribed more than one class in a given year they are represented in both classes. Where a child is prescribed two or more classes this may indicate polypharmacy or a switch to an alternative medication

^c^Total children receiving prescriptions of a given class over all study years

^d^Total children receiving prescriptions in a given year

**Table 3:** Number of children prescribed psychotropic classes per year—K cohort (N = 157)

Psychotropic Classes^b^	Age range (yrs)^a^/Year																		Total children^c^
	2–3	4–5		6–7		8–9		10–11		12–13		14–15		16–17		18–19		20–21	
	2002	2003	2004	2005	2006	2007	2008	2009	2010	2011	2012	2013	2014	2015	2016	2017	2018	2019	
Anticonvulsants	0	0	1	2	2	2	3	3	4	4	5	5	4	4	3	3	4	3	11
Antidepressants	1	1	3	1	3	3	6	7	13	13	14	18	20	21	24	32	30	20	66
Antipsychotics	0	0	1	3	2	8	11	12	16	12	11	13	12	15	9	13	9	5	37
Anxiolytics	1	0	0	0	0	0	0	0	0	0	0	0	2	0	2	3	1	0	7
Hypnotics	0	0	0	0	1	0	0	0	0	0	1	1	1	1	0	1	1	1	6
Clonidine	1	0	1	2	2	3	4	4	5	6	5	6	2	3	3	2	1	1	13
ADHD medications	1	1	1	3	12	16	21	21	18	23	24	21	18	15	12	8	9	5	43
Polypharmacy	1	0	1	3	5	8	10	9	16	19	19	23	14	19	13	15	13	8	50
Total children^d^	2	2	6	7	17	22	33	37	39	38	37	40	41	40	38	43	39	26	92

^a^Approximate ages are extrapolated from LSAC recruitment parameters, namely; recruitment occurred between 2003 and 2004 with the K cohort being comprised of preschoolers between 4 and 5 years

^b^If a given child is prescribed more than one class in a given year they are represented in both classes. Where a child is prescribed two or more classes this may indicate polypharmacy or a switch to an alternative medication

^c^Total children receiving prescriptions of a given class over all study years

^d^Total children receiving prescriptions in a given year

**Fig. 2:**
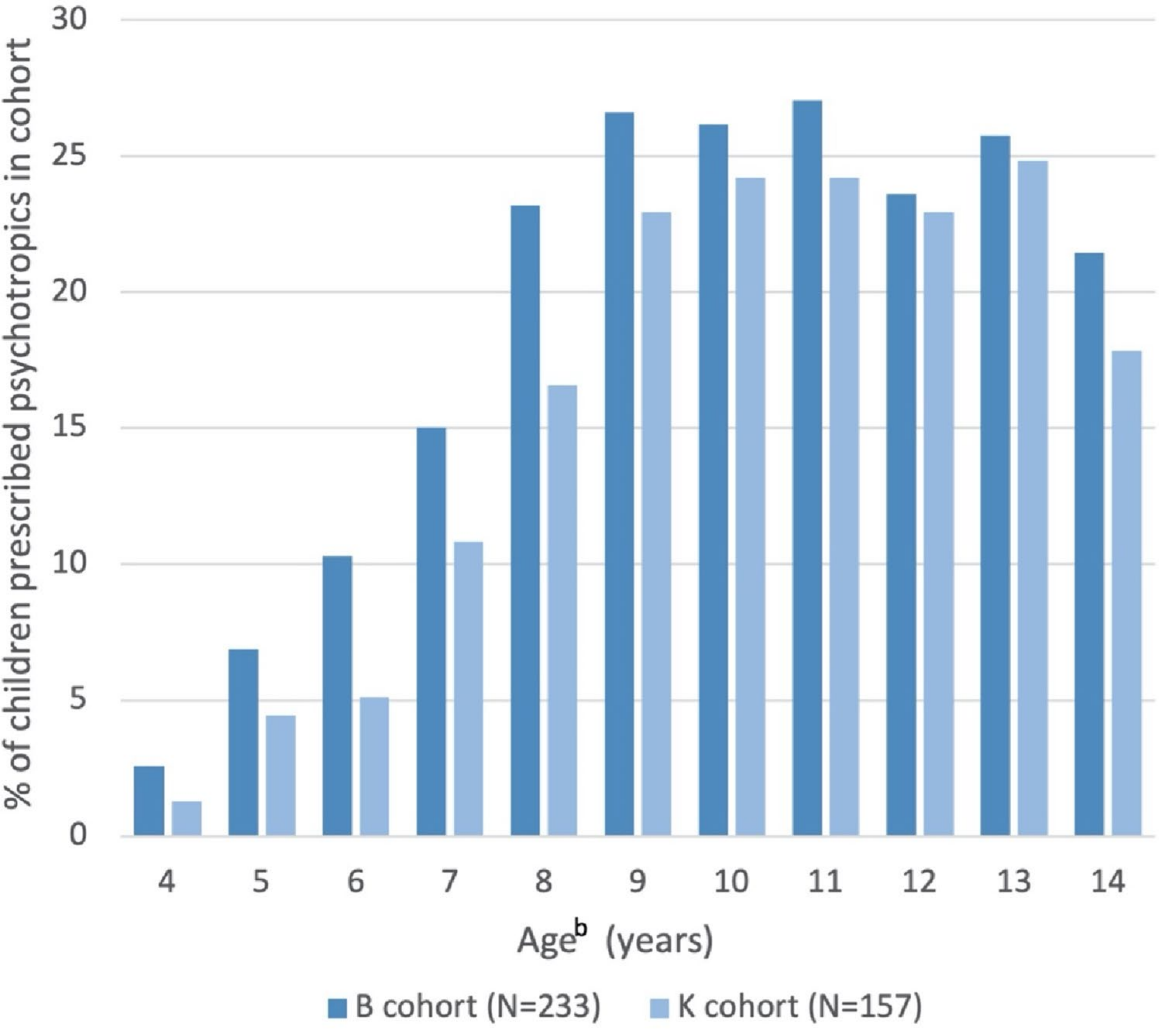
Autistic children and adolescents prescribed psychotropic agents^a^. ^a^Prescriptions limited to those supplied between 4 and 14 years old. ^b^Ages rounded to the closest integer

Limiting to prescriptions supplied between 4 and 14 years of age to allow for comparison between cohorts, there was a trend towards more children in the B cohort receiving a psychotropic prescription (47.2%) compared to the K cohort (37.6%); χ^2^ = 3.54, p = 0.06. The percentages of children prescribed a psychotropic agent over time is illustrated in Fig. 2. The median age of a psychotropic medication being first prescribed for the B cohort was 8 years and 4 months (IQR = 7–10 years) and K cohort 8 years and 5 months (IQR = 6 years and 9 months—10 years and 2 months). The median age of first polypharmacy prescription for the B cohort was 9 years (IQR = 6 years – 11 years and 7 months) and K cohort 10 years and 7 months (IQR = 7 years and 4 months – 11 years and 9 months). There was no significant difference between cohorts in the age of first psychotropic or polypharmacy prescription, p = 0.67 and 0.26 respectively. There was no difference in percentage of children prescribed polypharmacy, ADHD medications or anticonvulsants between the cohorts; p = 0.76, 0.42, and 0.76 respectively.

### Antidepressant Prescribing

In total 122 or 31.3% of children received at least one antidepressant prescription during the study period. The number of children prescribed a given antidepressant agent per year is presented in Table 4 and 5, for B and K cohorts respectively. The number of children prescribed antidepressants tended to increase with increasing age. Fluoxetine was the most frequently prescribed antidepressant throughout the study period, being prescribed in 57.1% (32/56) of the B cohort and 65.2% (43/66) of the K cohort who received an antidepressant prescription.

**Table 4:** Number of children prescribed antidepressants per year—B Cohort (N = 233)

Antidepressant^b^	Age range (yrs)^a^/Year																		Total children^c^
		0–1		2–3		4–5		6–7		8–9		10–11		12–13		14–15		16–17	
	2002	2003	2004	2005	2006	2007	2008	2009	2010	2011	2012	2013	2014	2015	2016	2017	2018	2019	
Amitriptyline	N/A	0	0	0	0	1	1	1	1	1	2	2	4	2	2	4	0	1	9
Citalopram	N/A	0	0	0	0	0	0	1	0	0	0	0	0	0	0	0	0	0	1
Desvenlafaxine	N/A	0	0	0	0	0	0	0	0	0	0	0	0	0	0	0	1	1	1
Duloxetine	N/A	0	0	0	0	0	0	0	0	0	0	0	0	0	0	0	0	0	0
Escitalopram	N/A	0	0	0	0	0	0	1	0	0	0	0	1	1	1	2	3	1	4
Fluoxetine	N/A	0	0	0	0	0	0	2	2	4	6	8	8	10	12	15	16	12	32
Fluvoxamine	N/A	0	0	0	0	0	0	0	0	1	1	1	3	3	4	5	4	2	11
Imipramine	N/A	0	0	0	0	0	1	0	0	1	1	3	3	1	0	0	0	0	4
Mirtazapine	N/A	0	0	0	0	0	0	0	0	0	0	0	0	0	0	0	1	0	1
Nortriptyline	N/A	0	0	0	0	0	0	0	0	0	0	0	0	0	0	0	0	0	0
Paroxetine	N/A	0	0	0	0	0	0	0	0	0	0	0	0	0	0	1	0	0	1
Reboxetine	N/A	0	0	0	0	0	0	0	0	0	0	0	0	0	0	0	0	0	0
Sertraline	N/A	0	0	0	0	0	0	0	0	2	1	1	1	2	2	4	8	6	15
Venlafaxine	N/A	0	0	0	0	1	0	0	0	0	1	0	0	0	0	0	0	0	2
Total children^d^	N/A	0	0	0	0	2	2	5	3	8	10	14	17	19	20	28	29	23	56

^a^Approximate ages are extrapolated from LSAC recruitment parameters, namely; recruitment occurred between 2003 and 2004 with the B cohort being comprised of infants between 0 and 1 years

^b^If a given child is prescribed more than one class in a given year they are represented in both classes. Where a child is prescribed two or more classes this may indicate polypharmacy or a switch to an alternative medication

^c^Total children receiving prescriptions of a given class over all study years

^d^Total children receiving prescriptions in a given year

**Table 5:** Number of children prescribed antidepressants per year—K Cohort (N = 157)

Antidepressant^b^	Age range (yrs)^a^/Year																		Total children^c^
	2–3	4–5		6–7		8–9		10–11		12–13		14–15		16–17		18–19		20–21	
	2002	2003	2004	2005	2006	2007	2008	2009	2010	2011	2012	2013	2014	2015	2016	2017	2018	2019	
Amitriptyline	1	0	0	0	0	0	1	1	2	1	0	0	0	1	1	0	1	0	6
Citalopram	0	0	0	0	0	0	0	0	0	0	0	0	1	1	1	2	3	3	5
Desvenlafaxine	0	0	0	0	0	0	0	0	0	1	0	0	0	1	0	1	1	2	4
Duloxetine	0	0	0	0	0	0	0	0	0	0	0	0	0	1	0	0	1	0	2
Escitalopram	0	0	0	0	0	0	0	2	1	1	1	1	2	0	2	6	4	2	14
Fluoxetine	0	0	2	1	1	2	4	4	8	9	11	16	12	14	13	17	13	6	43
Fluvoxamine	0	0	0	0	0	0	0	0	1	0	1	0	0	0	0	0	0	1	3
Imipramine	0	0	0	0	1	0	1	0	2	1	0	0	0	0	0	0	0	0	3
Mirtazapine	0	0	0	0	0	1	1	1	1	0	0	0	0	1	1	2	2	2	4
Nortriptyline	0	0	0	0	0	0	0	0	0	0	0	0	0	0	0	1	0	0	1
Paroxetine	0	0	0	0	0	0	0	0	0	0	0	0	2	1	1	2	2	1	4
Reboxetine	0	0	0	0	0	0	0	0	0	0	0	0	0	0	0	0	0	0	1
Sertraline	0	1	1	0	1	0	0	1	1	1	2	1	3	7	7	6	7	4	16
Venlafaxine	0	0	0	0	0	0	0	0	0	0	0	0	0	0	0	2	2	1	3
Total children^d^	1	1	3	1	3	3	6	7	13	13	14	18	20	21	24	32	30	20	66

^a^Approximate ages are extrapolated from LSAC recruitment parameters, namely; recruitment occurred between 2003 and 2004 with the K cohort being comprised of preschoolers between 4 and 5 years

^b^If a given child is prescribed more than one class in a given year they are represented in both classes. Where a child is prescribed two or more classes this may indicate polypharmacy or a switch to an alternative medication

^c^Total children receiving prescriptions of a given class over all study years

^d^Total children receiving prescriptions in a given year

**Fig. 3:**
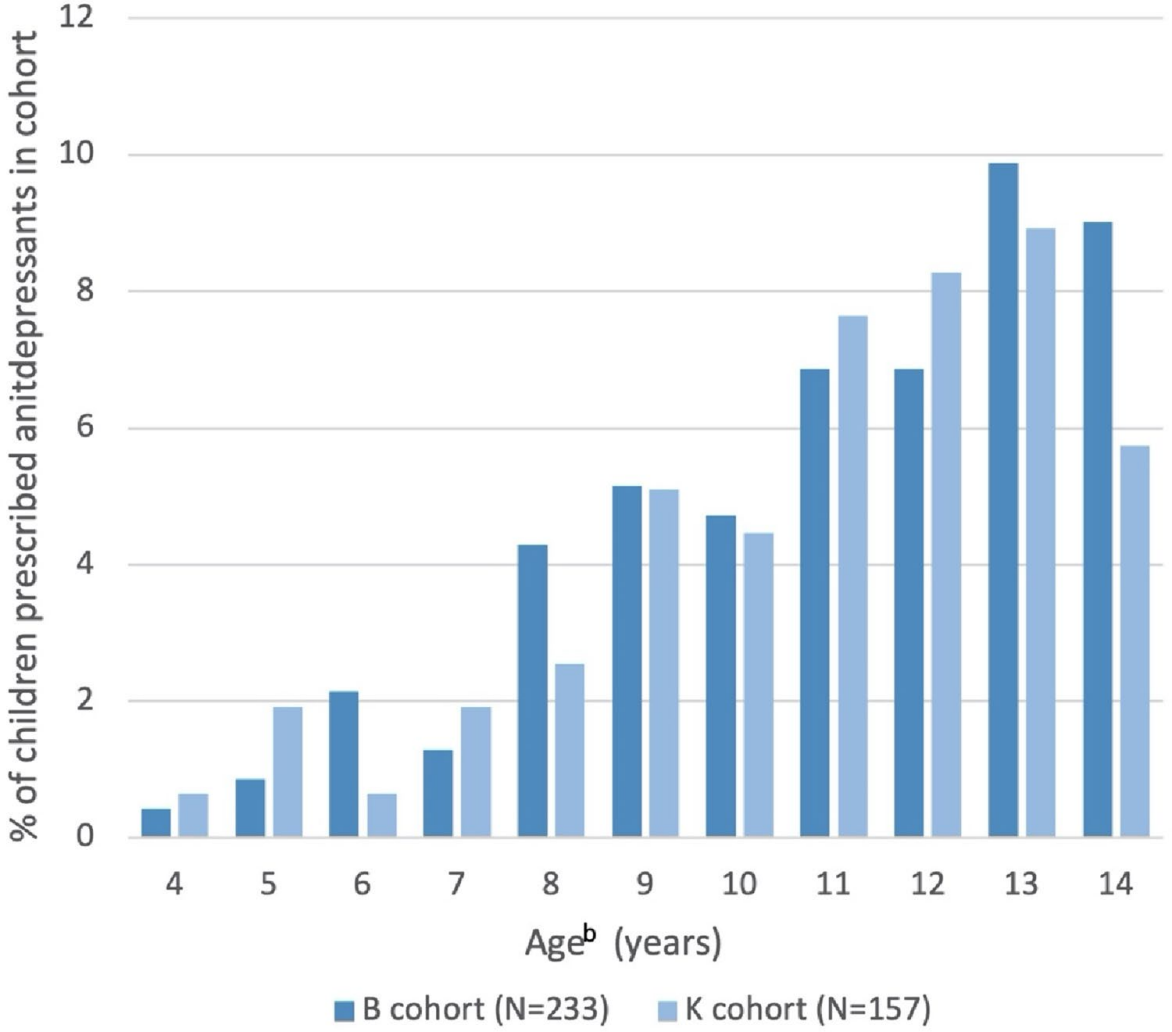
Autistic children and adolescents prescribed antidepressant agents^a^. ^a^Prescriptions limited to those supplied between 4 and 14 years old. ^b^Ages rounded to the closest integer

**Fig. 4:**
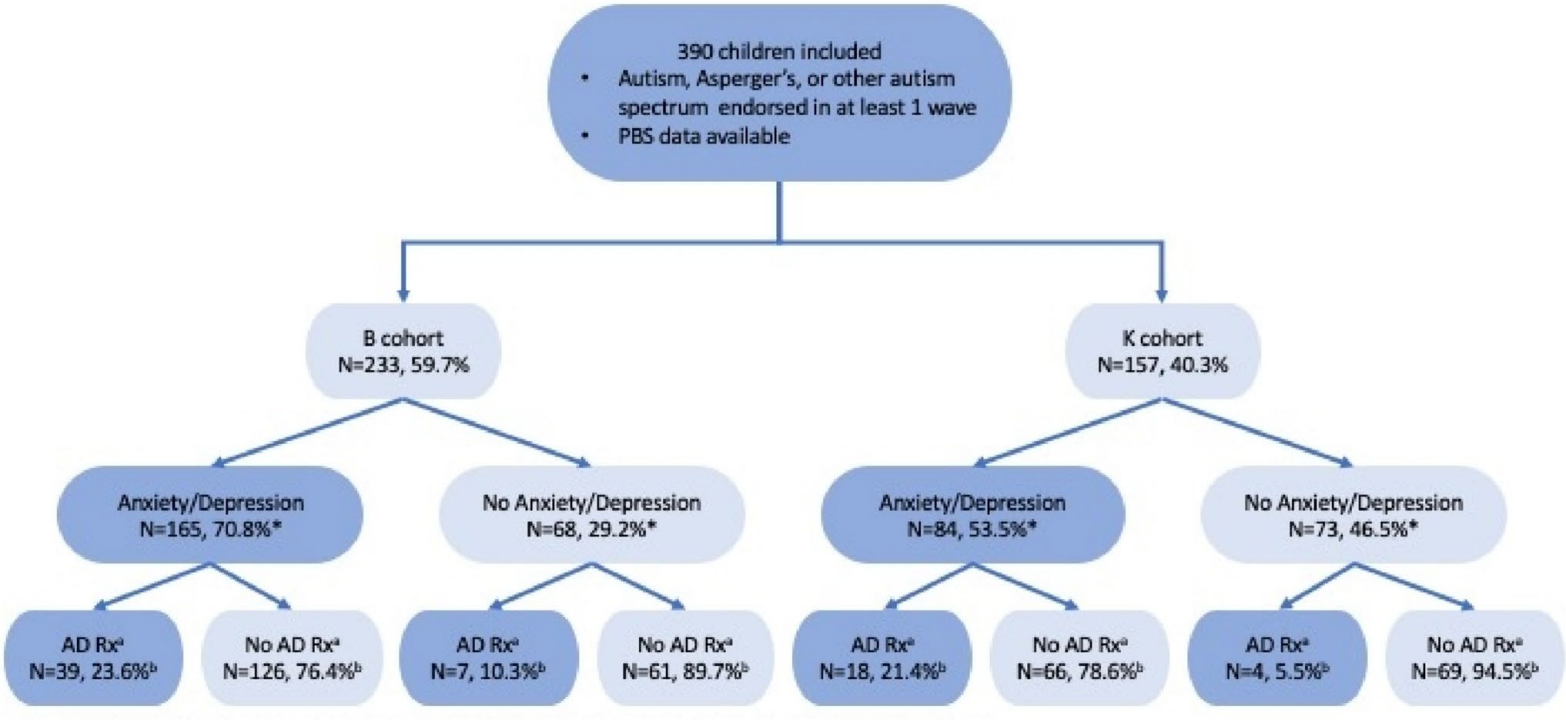
Antidepressant prescribing in relation to presence of diagnosis of anxiety or depression. *A diagnosis of anxiety or depression was endorsed more frequently by the B cohort than the K cohort; chi-square 12.18, p < 0.0005. ^a^AD Rx – antidepressant prescription. ^b^Prescriptions limited to those supplied between 4 and 14 years old to allow comparison between cohorts

**Fig. 5:**
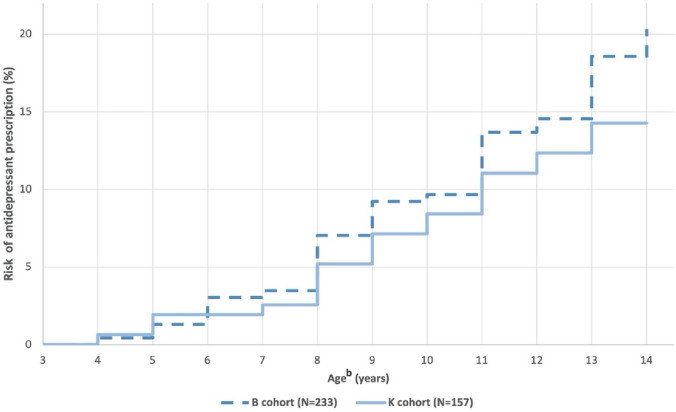
Survival curve to first antidepressant prescription^a^. ^a^Analysis limited to children or adolescents who received an antidepressant agent between 4 and 14 years. ^b^Ages rounded to the closest integ

Limiting to prescriptions supplied between 4 and 14 years of age to allow for comparison between cohorts, 46 or 19.74% children from the B cohort and 22 or 14.01% of children from the K cohort received at least one antidepressant prescription (p = 0.14). The percentages of children prescribed an antidepressant over time is illustrated in Fig. 3. The relation between children prescribed antidepressants and diagnosis of depression or anxiety disorder is illustrated in Fig. 4. Of children with a co-occurring diagnosis of anxiety or depression 39 children (23.6%) in the B cohort and 18 children (21.4%) in the K cohort received an antidepressant prescription. Where there was no diagnosis of anxiety or depression, antidepressants were prescribed in 7 children (10.3%) of the B cohort and 4 children (5.5%) of the K cohort. Fluoxetine was the first antidepressant agent prescribed for 47.1% of children (45.7% and 50.0% for B and K cohort, respectively), which was consistent between cohorts (p = 0.74). The median age of children first being prescribed an antidepressant was 10 years and 8 months (IQR = 8 years 1 month—12 years 7 months) for the B cohort, and 9 years and 10 months (IQR = 7 years 8 months—11 years 3 months) for the K cohort; with no significant difference between cohorts (p = 0.42). A survival curve illustrating the time to (equivalent to age) of first antidepressant prescription is provided in Fig. 5.

Most (76.1%) children in the B cohort and all children in the K cohort had a reported diagnosis of autism prior to receiving an antidepressant prescription (p = 0.09). The timeframe between autism diagnosis and first prescription of an antidepressant was on average 3 years and 4 months (SD = 3 years 11 months; range 6 years and 10 months prior to 12 years and 9 months post autism diagnosis) with no significant difference between cohorts (p = 0.21).

## Discussion

Psychotropic prescribing in autistic children is common with more than half of the children in this Australian population representative sample being prescribed at least one agent over the study period. High frequency of prescribing is consistent with previous studies, including one prior study using the same cohort and definition of classes but at a younger age [1, 2]. This study extends on that work by exploring prescribing over time, at an older age and exploring cohort differences. As expected, the present study’s use of a broader age range saw a higher frequency of prescribing consistent with the longer time period and increasing prescriptions as children become older.

High frequency of antidepressant prescribing in almost a third of autistic children is consistent with existing global evidence [1, 11]. Despite the present study only including children and adolescents, we found similar antidepressant prescribing rates to those that included participants over the entire lifespan; namely 31.3% compared to up to 38.8% in Alfageh et al.’s [11] study and up to 43% in one global systematic review [1]. More children in the B cohort were found to have a diagnosis of depression or anxiety. This may suggest the presence of a cohort effect potentially reflecting the increasing prevalence of anxiety and depression in young people seen in the general population [21] which has been suggested to be driven by decreased stigma and increasing awareness relating to the presence and impact of depression and anxiety in children, as well as, increasing prevalence of distress due to the impact of social media exposure, increasing access to news related to global crises, and increasing awareness of health, climate and financial issues [22, 23]. This was not, however, reflected in antidepressant prescribing frequency, with there being no difference between cohorts in percentages of children with an antidepressant prescription. Additionally, there was no difference between cohorts with regards to the average age of first antidepressant prescription suggesting that prescribing is not occurring at increasingly younger ages, and, there were a high percentage of autistic children with co-occurring anxiety and depression who were not medicated. These findings align with guidelines specifying the use of non-pharmacological strategies as first-line or for low symptom severity in anxiety and depression [24, 25]. This consists of psychoeducation, lifestyle changes and psychological therapies, with the greatest evidence being for cognitive behaviour therapy. However, it could also indicate that some children with autism and co-occurring anxiety and depression may not be having these secondary conditions treated, noting the evidence for SSRIs particularly in anxiety disorders [26, 27] and, when combined with psychotherapy, in moderate to severe depression [28]. There were only 11 autistic children (2.8%) without co-occurring anxiety and depression indicated who received antidepressant medication, which suggests reasonable awareness of evidence regarding ineffectiveness of antidepressants for repetitive restrictive behaviours or core symptoms of autism [14, 29, 30]. However, given that prescribing for these indications would be considered off label these prescriptions are likely not captured in the available PBS data hence it is difficult to draw conclusions regarding the prevalence of this practice.

When selecting an antidepressant agent, international and Australian evidence and guidance supports the use of fluoxetine as the first-line agent in the general population of children and adolescents with depression [15, 31, 32]. Though fluoxetine was the most frequently prescribed agent, it was used as the initial antidepressant in less than half of the children and adolescents receiving an antidepressant included in the study. This may reflect the use of antidepressants for anxiety disorders where guidance is less prescriptive; individualised prescribing taking into account contraindications and preferable side effect profiles; or a lack of awareness regarding current best practice. Given existing evidence for better tolerability and efficacy with fluoxetine in the general population of children and adolescents [15], this finding may indicate an area where targeted education could improve outcomes. However, caution is also needed when applying available guidelines given the low quality of the evidence on which these are based. The generalisability of the available evidence is limited by the heterogenous and small samples, limited follow up and lack of trials looking specifically at efficacy and tolerability in autistic or neurodivergent children and adolescents. Given the inadequacies inherent in available guidance and the unique complexities represented by this cohort, it is understandable that clinicians find themselves prescribing outside of existing guidelines.

### Strengths and Limitations

As with other studies based on LSAC, the key strength of this study is the availability of detailed longitudinal data including demographic and health information based on a population sample. The linkage with prescription data from PBS avoids recall bias, whilst providing a detailed longitudinal view of prescribing over the study period. Previous studies, utilising this data have focussed on particular timeframes, whilst the present study incorporated data from all available waves and from across the prescribing period that PBS data were available.

The main limitation of this study is the use of parent-report for the identification of children and adolescents with autism and co-existing diagnoses. We included all children where a diagnosis of autism was endorsed in the available LSAC data, even where information related to autism was incomplete, such as severity and age of diagnosis, which may further increase the risk of including non-validated data. Previous studies have excluded these children citing that where information related to autism was incomplete, data on other psychiatric and neurological conditions were also incomplete [2]. However, parent reported rates of autism have historically been similar to surveillance data [33] and additionally capture parent’s lived experience and perception of strengths and difficulties.

Though the PBS data used is robust, prescriptions can also be accessed privately in Australia, hence these findings may underestimate the actual psychotropic use in autistic children. Any medication accessed by these means would not be captured. It is unclear how commonly medications are accessed in this way. However, private prescribing may represent instances of off-label use, which may be relevant given the limited indication for medications in this population. Conversely, although medications are prescribed they may not be taken.

## Conclusions

Over half the autistic children in an Australian sample received at least one prescription of a psychotropic agent between 2002 and 2019, whilst over a quarter had polypharmacy. As expected, psychotropic prescribing increased with increasing age. More than half of children endorsed a co-occurring diagnosis of anxiety or depression, with significantly higher prevalence in the younger B cohort suggestive of a cohort effect which requires further exploration. Likely reflective of the high frequency of co-occurring depression or anxiety and similar to previous studies, antidepressants were the most commonly supplied psychotropic agents. In line with clinical guidelines recommending non-pharmacological interventions as first-line, the majority of autistic children with anxiety or depression were however not prescribed an antidepressant agent. Rates of off label prescribing of antidepressants for the core symptoms of autism are not captured in the present study as it relies on PBS prescription data. Converse to existing guidelines and existing evidence, fluoxetine was the first-line antidepressant in less than half of the included children and adolescents, representing a potential area for targeted education. Australian prescribing guidelines for autism are urgently needed with further studies looking at outcomes and tolerability to inform these.

## Summary

Medications have not been shown to be effective in changing core autistic social communication characteristics. However, psychotropic agents are commonly used to support psychological symptoms and co-occurring conditions; including severe behavioural disorders, attention-deficit/hyperactivity disorder (ADHD), anxiety and depression. As such psychotropic prescribing in autistic children is common. The heterogeneity that exists both demographically and clinically in autistic children is reflected in the diversity of prescribing and makes it difficult to develop prescribing guidelines. With the view to help inform future guidelines, we therefore described the utilisation of psychotropic medications in autistic children in Australia using linked data from The Longitudinal Study of Australian Children (LSAC) waves 1 to 9 and Pharmaceutical Benefits Scheme (PBS) between 2002 and 2019. We included the 390 children, consisting of 233 children from the B cohort (age 0–1 years in wave 1) and 157 children from the K cohort (age 4–5 years in wave 1), who endorsed a diagnosis of autism. We found over half of autistic children received at least one prescription of a psychotropic agent between 2002 and 2019, whilst over a quarter had polypharmacy. As expected, psychotropic prescribing increased with increasing age. More than half of children endorsed a co-occurring diagnosis of anxiety or depression, with significantly higher prevalence in the younger B cohort suggestive of a cohort effect which requires further exploration. Likely reflective of the high frequency of co-occurring depression or anxiety and similar to previous studies, antidepressants were the most commonly supplied psychotropic agents. In line with clinical guidelines recommending non-pharmacological interventions as first-line, the majority of autistic children with anxiety or depression were however not prescribed an antidepressant agent. Rates of off label prescribing of antidepressants for the core symptoms of autism are not captured in the present study as it relies on PBS prescription data. Converse to existing guidelines and existing evidence, fluoxetine was the first-line antidepressant in less than half of the included children. This may reflect the use of antidepressants for anxiety disorders where guidance is less prescriptive; individualised prescribing taking into account contraindications and preferable side effect profiles; or a lack of awareness regarding current best practice and a potential area for targeted education. Australian prescribing guidelines for autism are urgently needed with further studies looking at outcomes and tolerability to inform these.

## Data Availability

Confidential unit record files from the LSAC survey are used which can be accessed through application and registration with LSAC via: https://growingupinaustralia.gov.au/data-and-documentation/accessing-lsac-data.
